# Comprehensive Characterization and Comparison of Aroma Profiles of *Tricholoma matsutake* Soup During the Cooking Process by HS-GC-IMS and HS-SPME-GC-MS

**DOI:** 10.3390/foods14091478

**Published:** 2025-04-24

**Authors:** Ni-Na Yan, Cheng-Jian Xu, Shuang-Li Xiong, Xiang Li, Si-Yu Gu, Zhong-Yan Zhu, Yang Liu, Nan Zhu, Yu Zhou, Hang Xiao

**Affiliations:** 1 Key Laboratory of Sichuan Cuisine Artificial Intelligence, Sichuan Tourism University, Chengdu 610100, China; yannina_yfy@163.com (N.-N.Y.); 0001427@sctu.edu.cn (S.-L.X.); shiclx@163.com (X.L.); siyu6222@163.com (S.-Y.G.); zhuzhongyan123@163.com (Z.-Y.Z.); yangliuy521l@163.com (Y.L.); 13880651735@sohu.com (N.Z.); cherryzhouyu119@163.com (Y.Z.); 2 College of Culinary and Food Science Engineering, Sichuan Tourism University, Chengdu 610100, China; 3 Department of Food Science, University of Massachusetts, Amherst, MA 01003, USA; hangxiao@foodsci.umass.edu

**Keywords:** *Tricholoma matsutake*, HS-GC-IMS, HS-SPME-GC-MS, ROAV, volatile compounds

## Abstract

Many scholars have studied *Tricholoma matsutake* soup, but there are relatively few studies exploring the aroma changes during its cooking process using different detection methods. The aroma of *T. matsutake* soup was analyzed and compared using electronic nose (E-nose) analysis, headspace gas chromatography-ion mobility spectrometry (HS-GC-IMS), and headspace-solid-phase microextraction-gas chromatography-mass spectrometry (HS-SPME-GC-MS). A significant effect of cooking time on the overall aroma profile of *T. matsutake* soup was identified through E-nose analysis. By HS-GC-IMS and HS-SPME-GC-MS analysis, 51 volatile aroma compounds were detected, with alcohols and aldehydes identified as the main aroma substances. Based on the relative odor activity value (ROAV) and multivariate statistical analysis, 1-octen-3-ol, 1-octanol, methyl cinnamate, and 2-pentyl furan were determined to be the key aroma compounds in the cooking process. We observed that shorter cooking time preserved the mushroom aroma of *T. matsutake* soup most effectively. These findings can be utilized for the industrial production of *T. matsutake* soup and for optimization of its key aroma components.

## 1. Introduction

*Tricholoma matsutake* is classified as a fungus in the phylum Basidiomycetes and the family *Tricholoma*. Its growth requires specific environmental conditions, and it is primarily distributed in high-altitude forest areas of the southwestern and northeastern regions of China. *T. matsutake* is highly valued for its rich nutritional content and unique, delicious aroma. Additionally, polysaccharides, dietary fiber, steroids, and polyphenols found in *T. matsutake* have been reported to exhibit biological activities, including immunomodulatory [1], anti-tumor [2], anti-oxidation [3], anti-fatigue [4], anti-inflammatory, wound healing, blood pressure-lowering, and antibacterial effects. It is widely used as an essential raw material for soup preparation.

Different cooking methods have been shown to affect the nutritional content and aroma compounds of edible mushrooms. In recent years, efforts have been made to optimize the cooking processes of edible mushrooms to maximize the retention of their nutrients and aromas. For example, slow roasting was found to significantly enhance the meat aroma intensity, overall acceptance, and umami of portobello mushrooms compared to untreated samples, while open-flame roasting did not show similar effects [5]. The roasting process was also reported to play a key role in generating aroma compounds in button mushrooms, which contribute to their unique taste [6]. Additionally, the volatile-component content of cooked *Agaricus bisporus* soup was significantly higher than that of steamed or microwave-cooked *A. bisporus* soup (*p* < 0.05) [7]. However, limited research has been conducted on the aroma profile of *T. matsutake* soup and the changes in its aroma during cooking.

HS-SPME-GC-MS is commonly used to detect volatile aroma compounds in food. This method combines the strengths of chromatography and mass spectrometry, providing high sensitivity and strong qualitative capability for qualitative and quantitative analysis of volatile compounds [8]. Similarly, headspace-gas chromatography-ion mobility spectrometry (HS-GC-IMS) is utilized for analyzing volatile aroma compounds in food as it offers an intuitive approach to assessing the volatile compounds in food samples comprehensively. HS-GC-IMS has the advantages of requiring no sample pretreatment, enabling rapid analysis, and achieving a low detection limit [9]. The combination of HS-SPME-GC-MS and HS-GC-IMS has been shown to overcome their respective limitations while leveraging their strengths. This combined approach expands the detection range of volatile components in samples and enhances the accuracy and reliability of analysis. However, no comprehensive study has yet been conducted on the characterization of volatile compounds in *T. matsutake* soup using both HS-SPME-GC-MS and HS-GC-IMS.

In this study, the overall aroma profile of *T. matsutake* soup was characterized using an E-nose, while the changes in volatile aroma compounds during the cooking process were analyzed by HS-GC-IMS and HS-SPME-GC-MS. Key aroma compounds in *T. matsutake* soup were identified based on relative odor activity values (ROAVs) and multivariate statistical analysis. This research aims to provide a theoretical basis for the aroma control and improvement of products related to *T. matsutake* soup, as well as a reference for the diversified utilization of *T. matsutake* and other edible fungi.

## 2. Materials and Methods

### 2.1. Preparation of T. matsutake Soup

Based on the preliminary test, the surface of fresh *T. matsutake* (Yunnan, China, 99.7078° E, 27.8231° N) was cleaned and the surface water was removed. The mushrooms were then cut into uniformly sized particles. Once the temperature had reached the predetermined value, *T. matsutake* was cooked in water at a material-to-liquid ratio of 1:60 (*w*/*v*, g/mL) at 100 °C for different cooking times (30, 60, 90, and 120 min; this process was repeated once, and the samples were labeled as A, B, C, and D, respectively).

### 2.2. E-Nose Analysis

An αFox4000 E-nose (Alpha M.O.S, Toulouse, France) equipped with 18 metal oxide semiconductor sensors was used to measure volatile compounds in a 10 mL sealed headspace bottle containing 2 mL of *T. matsutake* soup. Before sample collection, the samples were incubated at 50 °C for 15 min to reach equilibrium. Headspace injection was performed using an Alpha M.O. HS100 automatic sampler, with a sample volume of 1500 μL. Synthetic air served as the carrier gas, permanent airflow rate was 150 mL/s, and injection speed was 1500 μL/s. The data collection time was set to 120 s, while the total analysis time was set to 1080 s.

Each group of samples was tested in parallel five times, and a stable signal collected at 120 s was selected as the analysis data for three replicates. The response characteristics of each sensor in the αFox4000 E-nose are provided in Table 1. The higher the response value of the E-nose sensor, the stronger the aroma of the sample [10].

### 2.3. HS-GC-IMS Analysis

The analysis of *T. matsutake* soup samples was conducted using an HS-GC-IMS device (FlavourSpec^®^; Gesellschaft für Analytische Sensorsysteme GmbH, Dortmund, Germany). A 5 mL sample of *T. matsutake* soup was transferred into a 20 mL headspace injection bottle, sealed, and incubated at 80 °C for 15 min. After incubation, 0.5 mL of the sample was automatically injected through a heated syringe. The sample was propelled into an FS-SE-54-CB-1 column (15 m × 0.53 mm) using nitrogen gas (99.999% purity) as the carrier. The nitrogen carrier gas flow rate was programmed as follows: an initial flow rate of 2.0 mL/min was maintained for 2 min, which was then increased to 20.0 mL/min over 10 min, further increased to 100.0 mL/min from 10 to 20 min, and finally raised to 150.0 mL/min from 20 to 30 min. The analytes were subsequently eluted into the ionization chamber, where nitrogen gas with a flow rate of 150 mL/min (purity > 99.999%) served as the drift gas. Molecules were ionized in positive-ion mode by beta rays (tritium, 3H). The resulting ions were driven into a drift tube (98 mm in length), which was operated at a temperature of 45 °C and a voltage of 500 V/cm. Qualitative analysis of the compounds was performed by comparing their retention indices and drift times. Each group of samples was tested in parallel three times.

### 2.4. HS-SPME-GC-MS Analysis

The *T. matsutake* soups were analyzed using HS-SPME-GC-MS (Clarus^®^ SQ 8; Perkin Elmer, CT, USA). A 5 mL sample of *T. matsutake* soup was transferred into a 15 mL headspace bottle and equilibrated in a water bath at 80 °C for 20 min. The SPME fiber (2 cm) coated with aged DVB/CAR/PDMS was then inserted into the headspace bottle at 80 °C to adsorb volatile compounds for 40 min. Subsequently, the extracted SPME fiber was inserted into the gas chromatographic inlet for desorption at 250 °C for 10 min. The samples were propelled into an Elite-5MS column (30 m × 0.25 mm × 0.25 μm) using helium gas with a flow rate of 1 mL/min (99.999% purity). A split ratio of 5:1 was applied. The oven temperature program was as follows: the initial temperature was maintained at 40 °C for 3 min and then increased to 60 °C at 3 °C/min and held for 1 min; it was further increased to 140 °C at 6 °C /min and held for 1 min and finally increased to 250 °C at 20 °C/min and held for 2 min. When the analytes were eluted to the mass spectrometry detector, detection was carried out in electron ionization (EI) mode with an ionization energy of 70 eV, a source temperature of 230 °C, a quadrupole temperature of 250 °C, and a solvent delay of 1 min, and in full-scan acquisition mode (mass range: *m*/*z* 35–400).

The resulting chromatograms were analyzed based on peak area, retention time, spectra, and base peaks. Each peak was matched against the compound library provided by the National Institute of Standards and Technology (NIST). Compounds with a similarity index greater than 80 were considered to be present in the sample. The relative contents of volatile compounds in *T. matsutake* soup were quantified using the peak area normalization method [11].

### 2.5. ROAV Analysis

The contribution of each volatile aroma compound to the aroma of *T. matsutake* soup was evaluated using the ROAV parameter [12]. This parameter sets ROAVstan = 100 for the element contributing the most to each sample odor. For other components, the ROAV was computed as follows:

(1) $$ROAV_{A}\approx \frac{C{\%}_{A}}{C{\%}_{As\tan}}\times \frac{T_{s\tan}}{T_{A}}\times 100$$

where *C*%*_A_* represents the relative content of each volatile odor compound (%), *T_A_* represents the odor threshold of each volatile odor compound (mg/kg), *C*%*_A_*_stan_ represents the relative content of the component with the highest overall odor contribution in each sample (%), and *T_s_*_tan_ represents the odor threshold of the component with the highest overall odor contribution in each sample (mg/kg).

### 2.6. Data Analysis

The experimental results are expressed as mean ± standard deviation. Data normalization was performed using SPSS 27.0 (IBM Corp., Armonk, NY, USA). The radar map was generated using Origin 22.0 (Origin Lab Corporation, Northampton, MA, USA). Multivariate statistical analysis plots were created using SIMCA 14.1 (Umetrics AB, Umea, Sweden). Principal component analysis (PCA) was conducted at https://www.chiplot.online/. The clustering heat map, histogram, pie chart, and Venn diagram were created at https://www.omicstudio.cn/tool, which was accessed on 1 December 2024.

## 3. Results and Discussion

### 3.1. Analysis of E-Nose Results

**Figure 1:**
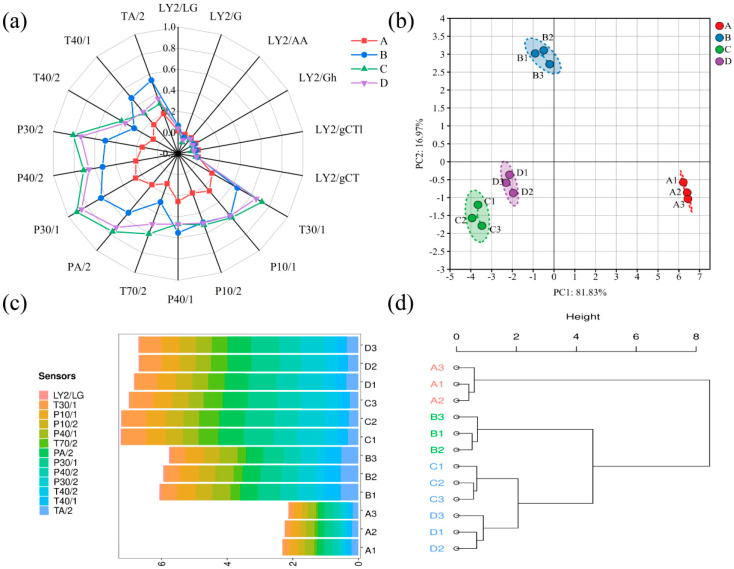
Response values for the E-nose sensor radar diagram for *T. matsutake* soup (**a**), PCA diagram (**b**), E-nose response value accumulation diagram (**c**), and HCA diagram (**d**). The data from three parallel experiments for each group of samples were analyzed. “A” means that the cooking time is 30 min, “B” means that the cooking time is 60 min, “C” means that the cooking time is 90 min, and “D” means that the cooking time is 120 min.

An E-nose is described as an instrument mimicking the function of biological olfactory systems, with the ability to detect and identify the composition and characteristics of complex odor mixtures [13]. The whole aroma profile of *T. matsutake* soup was analyzed using the E-nose. The response values of the samples in the E-nose sensors T30/1, T70/2, TA/2, P30/1, P30/2, and P40/2 were observed to be significantly higher than those of other sensors. This indicates that *T. matsutake* soup is characterized by relatively rich hydrocarbons, aromatic compounds, strongly oxidizing compounds, and polar compounds (Figure 1a). Moreover, sample C has the highest response value in the E-nose sensor, indicating that sample C has an aroma stronger than that of the other samples. According to the PCA results (Figure 1b), the cumulative contribution rate of the first and second principal components was calculated as 98.8%, covering most of the relevant information. This demonstrates that the PCA effectively captures the main change characteristics of the E-nose data. The four types of *T. matsutake* were effectively distinguished, and cooking time was shown to significantly impact the aroma of *T. matsutake*. Samples C and D were positioned in the third quadrant, at a relatively close distance, suggesting that the aroma profiles of these two samples may be similar. Based on the accumulation diagram of the E-nose response values (Figure 1c), the response value of *T. matsutake* was found first to increase and then decrease with the prolongation of the cooking time. This phenomenon may be attributed to the gradual release of aroma compounds from the fruiting bodies of *T. matsutake* into the soup during the initial cooking period, followed by volatilization of these compounds during extended cooking. Upon hierarchical cluster analysis (HCA) of the E-nose data, the four types of *T. matsutake* were classified into three categories: sample A as one category, sample B as another, and samples C and D as a third category. The similar aroma profiles of samples C and D are consistent with the findings of the PCA. The electronic nose can quickly distinguish the overall aroma profile, but it cannot identify specific compounds. It needs to be combined with GC technology for complementary analysis.

### 3.2. Difference Analysis of Volatile Components in T. matsutake Soup Cooking Process Based on HS-GC-IMS

**Figure 2:**
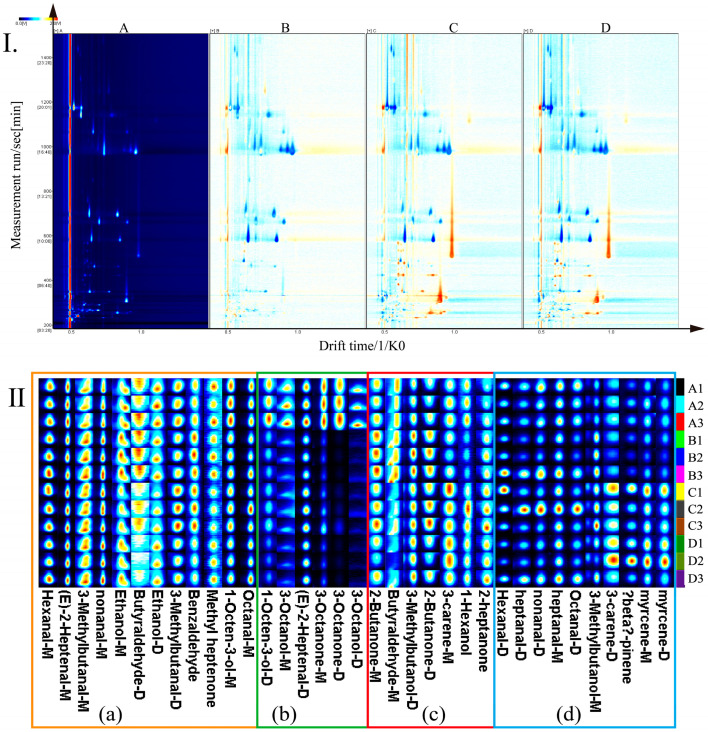
Comparison of HS-GC-IMS spectra of *T. matsutake* soup with different cooking times (**I**) and fingerprints (**II**).

The two-dimensional (2D) contrast spectrogram of HS-GC-IMS is a comparison diagram formed by subtracting the background of sample A, with blue representing low intensity and red representing high intensity (Figure 2I). Most of the signals in the atlas are in the 2D spectral region with a retention time of 200–1400 s, and the number of signals in the 2D spectrum is almost the same, but the peak signal intensity of each aroma substance is different. Compared with sample A, the concentration of most compounds with a retention time of 200–900 s in samples B, C, and D increased, and the concentration of most compounds with a retention time of 900–1400 s decreased. The cooking time affected the content of aroma substances in *T. matsutake* soup.

It can be seen from the fingerprints (Figure 2II) that 20 volatile aroma compounds were identified in *T. matsutake* soup, including some dimers of compounds, and that the content of compounds changed significantly with the prolongation of cooking time. When the proton affinity of the reactant exceeds that of a highly concentrated substance in water, the protons transfer to a substance with a higher affinity, thus catalyzing the formation of dimers [14]. The compounds in box (a) are bright red in color, with a high content, and the content changes little with the extension of cooking time. These compounds mainly include heptanal-*M*, *(E)*-2-heptenal-*M*, 3-methylbutanal-*M/D*, nonanal-*M*, octanal-*M*, and 1-octen-3-ol-*M*. The compounds in box (b) are present at high levels in sample A, and they mainly include 1-octen-3-ol-*D*, 3-octanol-*D*, and 3-octanone-*M/D*. (c) The content of compounds mainly including 2-butanone-*M*, butyraldehyde-*M*, 1-hexanol, 2-butanone-*M*, and 3-carene-*M* in the box is low, but the content changes little with the extension of cooking time. The content of compounds in box (d), which mainly include 3-bethylbutanol-*M*, beta-pinene, and myrcene-*M/D*, is high in the C and D samples, and the content gradually increases or first increases and then decreases with the extension of cooking time.

**Figure 3:**
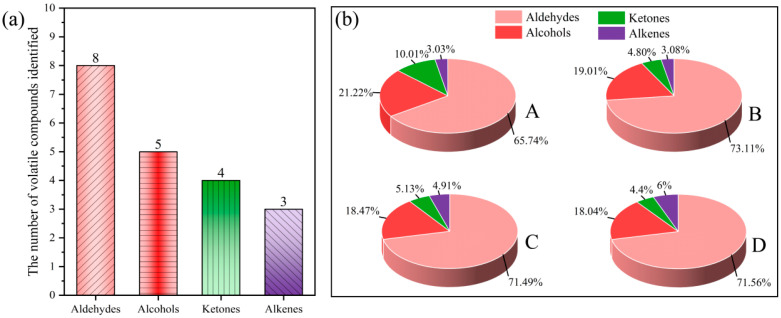
Column chart of volatile compounds quantity (**a**) and pie chart of relative content of volatile compounds types (**b**) in *T. matsutake* soup from HS-GC-IMS for different cooking times. “A” means that the cooking time is 30 min, “B” means that the cooking time is 60 min, “C” means that the cooking time is 90 min, and “D” means that the cooking time is 120 min.

The compounds identified in the samples were semi-quantitatively analyzed by a normalization method, and the relative contents of volatile aroma compounds in each sample were calculated (Figure 3 and Table 2). *T. matsutake* soup contains eight kinds of aldehydes, five kinds of alcohols, four kinds of ketones and three kinds of olefins. The main volatile aroma substances in *T. matsutake* soup were aldehydes (65.73–73.11%), followed by alcohols (18.04–21.22%), ketones (4.40–10.01%), and olefins (3.03–6.00%). The content of volatile aroma compounds changed significantly during the cooking process of *T. matsutake* soup.

The relative content of aldehyde compounds increased gradually with the extension of cooking time. Most aldehydes have been shown to be produced by lipid oxidation [15]. The relative contents of *(E)*-2-heptenal (almond, fat), nonanal (green, lemon), and octanal (fat) were high in the cooking process of *T. matsutake* soup. Nonanal and octanal may be produced by oxidation of linoleic acid, arachidonic acid, and oleic acid [16]. *(E)*-2-Heptenal is an unsaturated aldehyde that is probably produced by oxidation of n-6 polyunsaturated fatty acids.

The relative content of alcohol compounds did not change significantly during cooking. Alcohol compounds usually give food taste and sensations such as sweetness, fullness, aroma, and freshness. Linear alcohols are usually produced by lipid oxidation, whereas branched-chain alcohols are mainly produced by microbial degradation of branched-chain aldehydes [17]. Among them, 1-octen-3-ol (earth, mushroom) and 3-octanol (mushroom, oil) are C8 compounds, which can be formed by enzymatic degradation of esters [18]. This class of compounds is known to affect the taste of edible mushrooms. The relative contents of 1-octene-3-ol and 3-octanol decreased gradually with the extension of cooking time, indicating that the mushroom aroma of *T. matsutake* soup is lost after a long time of cooking.

The relative content of ketones showed a decreasing trend. Ketones are usually derived from lipid oxidation, Maillard reaction, and amino acid degradation [18], and they have a profound impact on the aroma characteristics of foods. 3-Octanone-*M* (butter, herb), which is relatively high in *T. matsutake* soup, is considered to be an important contributor to the aroma of cooked *T. matsutake* [19] and is produced by oxidative degradation of unsaturated lipids or non-enzymatic aminocarbonyl reactions.

Olefin compounds easily decompose into alkanes and alcohols, and the relative content of olefin compounds increases with the extension of cooking time. Among them, β-pinene has a pine aroma.

### 3.3. Difference Analysis of Volatile Components in T. matsutake Soup Cooking Process Based on HS-SPME-GC-MS

**Figure 4:**
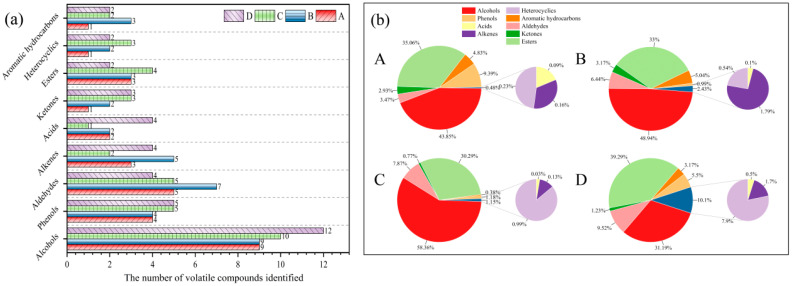
Column chart of compound quantity (**a**) and pie chart of relative content of compound species (**b**) in *T. matsutake* soup from HS-SPME-GC-MS for different cooking times. “A” means that the cooking time is 30 min, “B” means that the cooking time is 60 min, “C” means that the cooking time is 90 min, and “D” means that the cooking time is 120 min.

HS-SPME-GC-MS analysis was performed on *T. matsutake* soup in the cooking process, and the results are shown in Figure 4 and Table 3. A total of 59 volatile aroma compounds were identified in *T. matsutake* soup, among which alcohols (31.19–58.36%) and esters (30.29–9.29%) were the main volatile aroma compounds.

The relative content of alcohol compounds first increased and then decreased with the extension of cooking time. The precursor substances of alcohol compounds are mainly polyunsaturated fatty acids, a class of volatile aroma substances with a high threshold formed by lipid oxidation [20], which can make an important contribution to aroma when the concentration is high enough. The main components are 1-octen-3-ol (earth, mushroom), cedrol, and 2-propyl-1-heptanol, which are the main alcohols in *T. matsutake* soup.

As can be seen from Table 3, the relative content of aldehyde compounds in *T. matsutake* soup gradually increased with the prolongation of cooking time, same as the result of HS-GC-IMS. Aldehyde compounds such as benzaldehyde were mainly detected in *T. matsutake* soup. Benzaldehyde is an aromatic aldehyde with the aroma of bitter almond, caramel, and hyacinth. Long-chain fatty aldehydes, such as heptal, have a strong fruity aroma [21].

The relative content of ester compounds decreased first and then increased with the extension of cooking time, and the main component was methyl cinnamate (balsamic, strawberry). Ester substances are the main natural aroma suppliers, which can give *T. matsutake* soup a strong fruity aroma, which is generally produced by the non-enzymatic catalytic reaction of alcohol and organic acids or microbial enzymatic catalytic reaction [22]. The production of esters can significantly improve the astringent bitterness of the soup and enhance the overall aroma perception of *T. matsutake* soup. The highest relative content of methyl cinnamate can present cherry and aromatic ester aromas, and its production can significantly enrich the smell of *T. matsutake* soup.

Furan is derived from the oxidation of dehydrated carbohydrates and fatty acids or through the Amadori rearrangement mechanism, which mainly contributes to a caramel-like aroma [23], and 2-pentylfurann is oxidized and degraded from methyl linoleate [24]. Both compounds are phenol substances in *T. matsutake* soup; phenol and its derivatives have powerful aromatic components [25]. Phenolic substances interact with salivary proteins to produce astringency [26].

Ketones are mainly derived from the Maillard reaction or fat oxidation. Still, because of their low content and weak aroma in *T. matsutake* soup, they contribute little to the aroma of *T. matsutake* soup. The acid compounds are mainly produced by the hydrolysis and oxidation of fat in *T. matsutake* soup. Hydrocarbons generally have a high odor threshold and cannot directly give *T. matsutake* soup aroma, but they can be converted into other aroma compounds during high-temperature cooking.

### 3.4. ROAV Analysis of Volatile Components of T. matsutake Soup Based on HS-SPME-GC-MS

Because the compounds detected by HS-SPME-GC-MS were more diverse (51 species), covering a wider range of volatile aroma compounds in *T. matsutake* soup, they provided a larger data base for a comprehensive evaluation of the aroma contribution to *T. matsutake* soup. The core of ROAV analysis is evaluating the contribution of a particular compound to the overall aroma, and so the more types of compounds that are detected, the more comprehensive and accurate the analysis.

ROAVs of volatile aroma compounds detected by HS-SPME-GC-MS were analyzed to screen key aroma compounds (ROAV ≥ 1) in *T. matsutake* soup. As can be seen from Table 4, there are eight aroma substances with ROAV ≥ 0.1 that contribute to the aroma in *T. matsutake* soup samples. In the cooking process of *T. matsutake* soup, ROAVs of three compounds were always greater than 1, namely, 1-octen-3-ol (earth, mushroom), methyl cinnamate (balsamic, strawberry), and 2-pentylfuran (butter, floral, fruit). These three compounds are the key aroma compounds in *T. matsutake* soup. Among them, the ROAV of 1-octen-3-ol gradually decreased with the extension of cooking time, and the ROAV of 2-pentylfuran and methyl cinnamate showed an increasing trend, which indicates that long-term cooking resulted in loss of the mushroom aroma of *T. matsutake* soup, but it would bring some floral and fruity aroma.

Pentanal (bitter, oil) and nonanal (green, lemon) had ROAV ≥ 1 in samples B, C, and D and were the key odor components of *T. matsutake* soup samples with a cooking time of 30–120 min. In sample A, 0.1 ≤ ROAV ≤ 1. It can modify the aroma of *T. matsutake* soup [27]. With the extension of cooking time, the relative content of pentanal and nonanal in *T. matsutake* soup increased, and the contribution degree to the overall aroma of *T. matsutake* soup also increased gradually. The relative content of 1-octanol (bitter almond, fat) and ROAV decreased throughout the cooking process, and 1-octanol was lost over a long time. The value of benzaldehyde (bitter almond, burnt sugar, hyacinth-like) was 0.1 ≤ ROAV ≤ 1, indicating that it modified the aroma of *T. matsutake* soup.

### 3.5. Multivariate Statistical Analysis of Volatile Components of T. matsutake Soup Based on HS-SPME-GC-MS

**Figure 5:**
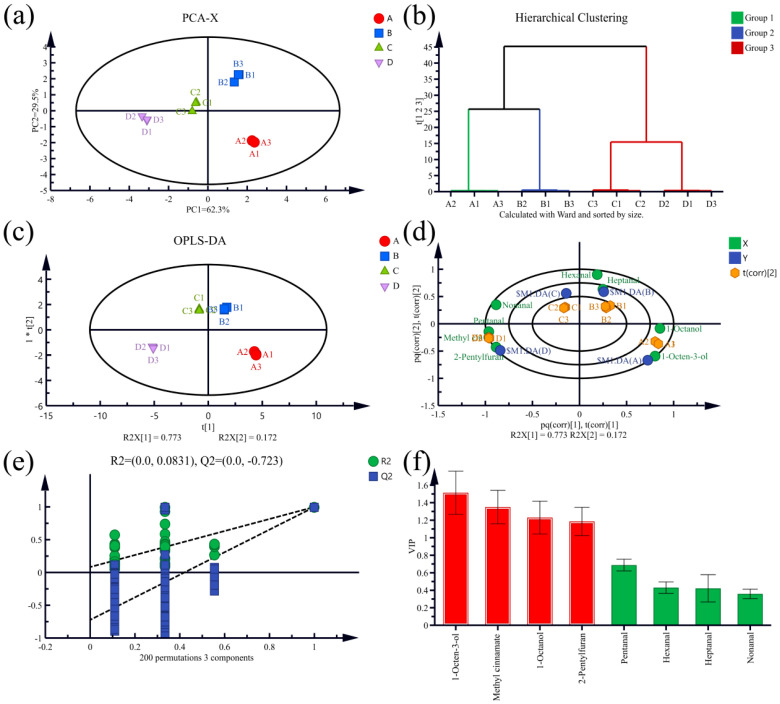
PCA diagram (**a**), HCA diagram (**b**), OPLS-DA diagram (**c**), biplot diagram (**d**), displacement test diagram (**e**), and VIP value diagram (**f**) from HS-SPME-GC-MS of *T. matsutake* soup compounds at different cooking times. In the (**f**) bar chart, red represents a VIP ≥ 1, and green represents a VIP value < 1.

Multivariate statistical analysis was performed for eight compounds with ROAV ≥ 1 identified by HS-SPME-GC-MS. According to PCA, the cumulative contribution rate of the first principal component and the second principal component is 91.8%, covering most of the information. It indicates that PCA can effectively capture the change characteristics of the data, and that the four kinds of *T. matsutake* soup can be effectively distinguished. HCA was used to identify the similarities and differences between samples. According to the characteristics of the data set, the samples were divided into three groups (Figure 5b). Sample C and sample D were divided into one group, indicating that the aromas of the two were similar, similar to the hierarchical clustering results of the E-nose. The OPLS-DA was further analyzed. In the OPLS-DA diagram, *T. matsutake* soup samples aggregated within the group and dispersed among the groups, and the R^2^X, R^2^Y, and Q^2^ values of the OPLS-DA model were 0.998, 0.998, and 0.996, respectively, indicating the stability and predictability of the model. In order to visualize the correspondence between aroma substances and different *T. matsutake* soups, a biplot of OPLS-DA was drawn. In the biplot (Figure 5d), the distance between the aroma substance and the *T. matsutake* soup sample represents the degree of correlation between the aroma substance and the *T. matsutake* soup samples. The closer the distance, the higher the correlation. 1-Octen-3-ol and 1-octanol were strongly correlated with sample A; and 2-pentylfuran, methyl cinnamate, and pentanal were strongly correlated with sample D. 1-Octen-3-ol (earth, mushroom) and 1-octen-3-ol (bitter almond, fat) were higher in sample A with cooking time of 30 min, and they were the main contributors to the aroma of sample A. The contents of 2-pentylfuran (butter, floral, fruit) and methyl cinnamate (balsamic, strawberry) were higher in sample D with cooking time of 150 min, and they were the main contributors to the aroma of sample D. The results of 200-fold displacement test (Figure 5e) prove the rationality and validity of the model. In the OPLS-DA model, according to the variable projected importance (VIP) values of each aroma component (Figure 5f), when the VIP value of a variable is >1, it is considered to be the key component of sample differentiation [28]. The aroma substances with outstanding contributions to *T. matsutake* soup that were selected included 1-octen-3-ol, 1-octanol, 2-pentylfuran, and methyl cinnamate (Table 5). From the analysis of ROAV, it can be seen that the contribution of 1-octen-3-ol and 1-octanol to the aroma of *T. matsutake* soup weakens with the extension of the boiling time, which shows that the “mushroom aroma” of *T. matsutake* soup gradually weakens with the extension of the cooking time. The ROAV of methyl cinnamate and 2-pentylfuran gradually increases with the extension of the cooking time to contribute to the flavor of *T. matsutake* soup, indicating that the floral and fruity aroma of *T. matsutake* soup is enhanced. Short-term cooking (30 min) may only release volatile substances on the surface of *T. matsutake*, while long-term cooking (90–120 min) may trigger lipid oxidation and Maillard reaction, producing more complex aroma compounds, but at the same time, it will lead to the loss of some mushroom characteristic aromas (such as C8 alcohols).

### 3.6. Combined Analysis by HS-GC-IMS and HS-SPME-GC-MS

**Figure 6:**
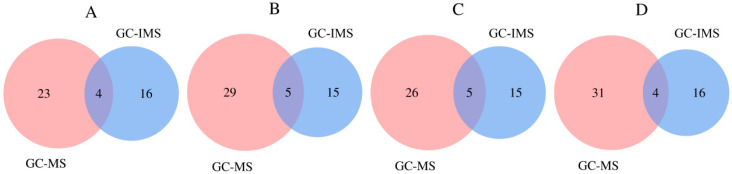
Venn diagram for HS-GC-MS and HS-SPME-GC-IMS results of aroma compounds in different *T. matsutake* soups. (**A**) means that the cooking time is 30 min, (**B**) means that the cooking time is 60 min, (**C**) means that the cooking time is 90 min, and (**D**) means that the cooking time is 120 min.

Only 20 to 35 volatile aroma compounds in *T. matsutake* soup were detected by a single method, while 43 to 51 volatile aroma compounds were detected by two methods (Figure 6). Among them, 1-octen-3-ol, 3-octanol, heptanal, 3-octanone, nonanal, hexanal, and benzaldehyde were detected at the same time in HS-GC-IMS and HS-SPME-GC-MS, indicating that these compounds were highly concentrated aroma compounds in *T. matsutake* soup and that they had important contributions to the aroma of *T. matsutake* soup.

HS-SPME-GC-MS is mainly based on the high resolution and sensitivity of mass spectrometry, which can isolate and characterize relatively high concentrations of compounds in samples. However, it may not be sensitive to some low-volatility or low-concentration compounds [29]. Some compounds may be decomposed or not fully volatilized under high-temperature gasification and column separation conditions of HS-SPME-GC-MS and therefore may not be detected by HS-SPME-GC-MS.

HS-GC-IMS is based on ion migration spectrometry technology and is more sensitive to some volatile compounds with polarity and low molecular weight, but it may be less effective in the detection of high-molecular-weight compounds or non-polar compounds in samples [29]. In addition, HS-GC-IMS is suitable for the analysis of highly volatile and easily ionized compounds, which means it may not effectively detect non-volatile or non-polar compounds.

The combination of the two methods can cover a wider range of volatile compounds: it merges the high resolution of HS-SPME-GC-MS with the high sensitivity of HS-GC-IMS to resolve the volatile aroma compounds of *T. matsutake* soup more comprehensively. HS-GC-IMS can be used to dynamically monitor the changes in volatile aroma compounds in *T. matsutake* soup during cooking. HS-SPME-GC-MS can accurately analyze the structure of the detected compounds and provide more detailed information for the study of the aroma mechanism of *T. matsutake* soup. This study comprehensively examined the chemical changes in volatile compounds in the cooking process of *T. matsutake* soup. Through the combination of the two methods, more compounds can be detected. Furthermore, it can be applied to the industrial production of *T. matsutake* soup, as well as the regulation and improvement of key aromas.

## 4. Conclusions

In this study, by combining electronic nose, HS-GC-IMS, and HS-SPME-GC-MS technologies, the aroma characteristics and dynamic changes in *T. matsutake* soup under different cooking times were comprehensively characterized. The research results show that the cooking time has a significant impact on the aroma profile of *T. matsutake* soup. A short cooking time (30 min) can retain the unique mushroom aroma of *T. matsutake* to the greatest extent, while a long cooking time (90–120 min) will lead to a weakening of the mushroom aroma and increase the content of floral and fruity compounds at the same time. Through ROAV analysis, it was determined that 1-octen-3-ol, 1-octanol, methyl cinnamate, and 2-pentylfuran were the key aroma substances of *T. matsutake* soup, and the contribution of these compounds to the overall aroma was particularly prominent. However, although long-term cooking can enrich the types of aroma compounds, it will lead to a significant reduction in the characteristic aromas of mushrooms (such as 1-octen-3-ol), which may affect consumers’ recognition of the traditional aroma of *T. matsutake* soup. Single analytical techniques (HS-SPME-GC-MS and HS-GC-IMS) have the problem of insufficient coverage when detecting volatile compounds, which may lead to the omission or misjudgment of some aroma substances. How to strike a balance between preserving the aroma of *T. matsutake* and developing diverse aromas is an urgent problem to be solved in the industrial production of *T. matsutake* soup.

This study, through the combination of multiple technologies and chemometrics analysis, not only provides a theoretical basis for the aroma regulation of *T. matsutake* soup but also offers a reference for the aroma research and technological improvement of other edible mushroom products. Future research can further explore the dynamic model during the boiling process, combine sensory evaluation and consumer preferences, and optimize the boiling process to achieve precise control of the aroma and quality improvement of *T. matsutake* soup.

## Figures and Tables

**Table 1:** Response characteristics and reference materials of 18 sensors of the E-nose.

Levels	Sensors Name	Response Characteristic	Reference Materials
1	LY2/LG	Sensitive to gases with strong oxidation capacity	Nitrogen oxides, sulfides, chlorine, fluorine
2	LY2/G	Sensitive to poisonous gases	Carbohydrates, amines, ammonia
3	LY2/AA	Sensitive to organic compounds	Ammonia, acetone, ethanol
4	LY2/GH	Sensitive to toxic gases	Amines, ammonia
5	LY2/gCTl	Sensitive to toxic gases	Sulphide
6	LY2/gCT	Sensitive to flammable gases	Butane, propane
7	T30/1	Sensitive to polar compounds	Propane, hydrogen chloride
8	P10/1	Sensitive to non-polar compounds	Carbohydrates, ammonia
9	P10/2	Sensitive to non-polar compounds	Methane, octane
10	P40/1	Sensitive to strong oxidizing gas	Chlorine and fluorine
11	T70/2	Sensitive to aromatic compounds	Toluene
12	PA/2	Sensitive to organic compounds	Ethanol, amines
13	P30/1	Sensitive to combustible gases and organic compounds	Hydrocarbons, ethanol, ammonia
14	P40/2	Sensitive to gases with strong oxidizing power	Sulfide, chlorine, fluoride
15	P30/2	Sensitive to organic compounds	Hydrogen sulfide, ketones
16	T40/2	Sensitive to gases with strong oxidizing capacity	Chlorine and fluorine
17	T40/1	Sensitive to gases with strong oxidizing capacity	Fluorine
18	TA/2	Sensitive to organic compounds	Ethanol

Note: P initials refer to plate-type sensors, and T initials refer to tube-type sensors. Both are based on a tin dioxide sensitive layer. LY sensors are plate type, with chromium titanium oxide and a tungsten oxide sensitive layer.

**Table 2:** Quantitative analysis and odor description from HS-GC-IMS of compounds in *T. matsutake* soup with different cooking times.

Compounds		Relative Content (%)				Odor Description *
	RI	A	B	D	E	
*(E)*-2-Heptenal-*D*	1291.6	8.54 ± 0.53 a	6.80 ± 0.91 b	7.69 ± 0.81 ab	7.38 ± 0.5 ab	Almond, fat
*(E)*-2-Heptenal-*M*	1293.5	9.87 ± 0.41 a	11.14 ± 1.42 a	10.32 ± 1.24 a	11.03 ± 0.51 a	Almond, fat
3-Methylbutanal-*D*	881.9	2.91 ± 0.07 a	3.20 ± 0.36 a	3.51 ± 0.40 a	3.22 ± 0.04 a	Almond, malt
3-Methylbutanal-*M*	880.6	1.57 ± 0.07 b	2.15 ± 0.24 ab	1.87 ± 0.22 a	2.05 ± 0.09 a	Almond, malt
Benzaldehyde	1511.5	0.95 ± 0.03 c	1.41 ± 0.22 a	1.21 ± 0.13 ab	1.10 ± 0.09 bc	Bitter almond
Butyraldehyde-*D*	845.6	0.07 ± 0.01 b	0.09 ± 0.01 a	0.06 ± 0.01 b	0.06 ± 0.01 b	Banana
Butyraldehyde-*M*	845.1	0.51 ± 0.02 b	0.81 ± 0.06 a	0.48 ± 0.12 b	0.41 ± 0.15 b	Banana
Heptanal-*D*	1166.2	0.54 ± 0.05 a	0.62 ± 0.22 a	0.73 ± 0.26 a	0.71 ± 0.13 a	Citrus, green
Heptanal-*M*	1165.7	2.30 ± 0.10 b	2.76 ± 0.25 a	2.90 ± 0.31 a	3.02 ± 0.20 a	Citrus, green
Hexanal-*D*	1055.2	1.48 ± 0.12 a	1.98 ± 0.58 a	2.08 ± 0.98 a	1.40 ± 0.30 a	Apple, fresh, green
Hexanal-*M*	1055.8	3.61 ± 0.01 b	4.81 ± 0.22 a	4.31 ± 0.56 a	4.24 ± 0.23 a	Apple, fresh, green
Nonanal-*D*	1393.6	8.08 ± 1.63 a	8.32 ± 3.58 a	8.68 ± 4.05 a	7.75 ± 1.37 a	Green, lemon
Nonanal-*M*	1393.6	16.39 ± 0.67 c	19.96 ± 0.79 a	17.86 ± 0.91 a	19.19 ± 0.30 b	Green, lemon
Octanal-*D*	1275.2	2.24 ± 0.24 a	1.85 ± 0.51 a	2.42 ± 1.01 a	2.31 ± 0.61 a	Citrus, fat
Octanal-*M*	1274.9	6.69 ± 0.30 a	7.22 ± 0.32 a	7.37 ± 0.56 a	7.69 ± 0.62 a	Citrus, fat
Aldehydes		65.73 ± 2.10 b	73.11 ± 2.86 a	71.49 ± 3.30 a	71.57 ± 2.71 a	-
1-Hexanol	1348.9	0.73 ± 0.06 bc	0.61 ± 0.10 c	0.90 ± 0.08 a	0.87 ± 0.08 ab	Banana, grass
1-Octen-3-ol-*D*	1452.7	2.17 ± 0.19 a	1.49 ± 0.24 b	1.46 ± 0.16 b	1.20 ± 0.07 b	Earth, mushroom
1-Octen-3-ol-*M*	1453	9.56 ± 0.42 a	9.21 ± 1.16 a	8.85 ± 1.09 a	8.46 ± 0.48 a	Earth, mushroom
3-Methylbutanol-*M*	1210.5	0.62 ± 0.05 b	1.04 ± 0.08 a	1.12 ± 0.18 a	1.04 ± 0.09 a	Cocoa, malt
3-Methylbutanol-*D*	1228.1	0.83 ± 0.05 b	1.30 ± 0.11 a	1.17 ± 0.14 a	1.30 ± 0.08 a	Cocoa, malt
3-Octanol-*D*	1398.9	2.35 ± 0.19 a	0.95 ± 0.09 b	0.92 ± 0.11 b	0.84 ± 0.05 b	Mushroom, oil
3-Octanol-*M*	1401.4	3.25 ± 0.28 a	2.10 ± 0.18 b	2.33 ± 0.18 b	2.05 ± 0.16 b	Mushroom, oil
Ethanol-*D*	895.7	1.07 ± 0.07 b	1.36 ± 0.16 b	1.06 ± 0.14 a	1.40 ± 0.11 a	Pungent
Ethanol-*M*	897.5	0.65 ± 0.04 b	0.95 ± 0.12 b	0.66 ± 0.08 a	0.88 ± 0.04 a	Pungent
Alcohols		21.22±1.18 a	19.01 ± 2.06 a	18.47 ± 1.73 a	18.04 ± 0.94 a	-
2-Butanone-*D*	867.2	0.55 ± 0.03 b	0.98 ± 0.12 a	0.89 ± 0.16 a	0.79 ± 0.08 a	Pleasant fruit
2-Butanone-*M*	865.5	0.49 ± 0.01 b	0.79 ± 0.07 a	0.61 ± 0.11 b	0.58 ± 0.10 b	Pleasant fruit
2-Heptanone	1162.7	0.41 ± 0.03 b	0.63 ± 0.06 a	0.63 ± 0.05 a	0.69 ± 0.01 a	Blue cheese, fruit, green
3-Octanone-*D*	1235	2.62 ± 0.20 a	0.18 ± 0.03 b	0.37 ± 0.21 b	0.19 ± 0.02 b	Butter, herb
3-Octanone-*M*	1236	5.58 ± 0.26 a	1.74 ± 0.29 b	2.25 ± 0.69 b	1.64 ± 0.02 b	Butter, herb
Methyl heptenone	1331.8	0.37 ± 0.02 b	0.48 ± 0.06 a	0.37 ± 0.02 b	0.49 ± 0.02 a	Citrus, Mushroom
Ketones		10.01 ± 0.52 a	4.80 ± 0.61 b	5.13 ± 1.22 b	4.40 ± 0.11 b	-
Beta-pinene	1098.9	0.33 ± 0.06 b	0.33 ± 0.05 b	0.60 ± 0.19 ab	0.81 ± 0.34 a	Pine
3-Carene-*D*	1134.5	0.49 ± 0.09 b	0.48 ± 0.09 b	0.87 ± 0.21 ab	1.06 ± 0.37 a	Lemon
3-Carene-*M*	1134	1.02 ± 0.12 b	1.11 ± 0.16 b	1.55 ± 0.35 ab	1.76 ± 0.40 a	Lemon
Myrcene-*D*	1179.1	0.54 ± 0.08 b	0.55 ± 0.10 b	0.89 ± 0.27 ab	1.17 ± 0.46 a	Fruits, herbs
Myrcene-*M*	1178.5	0.66 ± 0.08 b	0.61 ± 0.09 b	1.00 ± 0.25 ab	1.20 ± 0.38 a	Fruits, herbs
Alkenes		3.03 ± 0.43 b	3.08 ± 0.49 b	4.91 ± 1.27 ab	6.00 ± 1.94 a	-

Note: *M* represents the monomer of the compound and *D* represents the dimer of the compound. Each value is expressed as the mean ± SD (n = 3) of triplicate determinations. Significant differences between samples from different cooking times were analyzed using ANOVA. Means with different letters within a row are significantly different (*p* < 0.05) in different cooking times. RI: Retention index. The use of “-” indicates that the odor description of the compounds was not found. * Odor descriptions for volatile compounds were obtained from https://www.femaflavor.org/flavor-library (accessed on 12 February 2025).

**Table 3:** Quantitative analysis of compounds in *T. matsutake* soup by HS-SPME-GC-MS for different cooking times.

Compounds	RI	Relative Content (%)			
		A	B	C	D
2-(Methylamino)ethanol	8.32	0.05 ± 0.01 a	ND	0.15 ± 0.01 a	ND
1-Octanol	17.136	4.88 ± 0.04 ab	4.63 ± 0.05 c	0.45 ± 0.01 d	0.50 ± 0.01 a
Cubenol	23.025	1.30 ± 0.02	ND	ND	ND
1-Octen-3-ol	15.643	14.64 ± 0.11 a	4.73 ± 0.02 b	3.38 ± 0.07 c	3.17 ± 0.01 d
3-Octanol	14.917	0.30 ± 0.02 a	0.24 ± 0.02 b	0.24 ± 0.01 b	0.17 ± 0.01 c
4-Sec-Butylcyclohexanol	12.961	0.45 ± 0.01	ND	ND	ND
Cedrol	23.636	19.8 ± 0.02 c	22.26 ± 0.03 a	20.89 ± 0.01 b	16.45 ± 0.03 d
2-Isopropyl-5-methyl-1-hexanol	17.872	ND	1.82 ± 0.06 b	3.24 ± 0.11 a	ND
*(Z)*-2-Decen-1-ol	9.769	0.03 ± 0.01 c	0.07 ± 0.01 b	0.10 ± 0.01 a	0.03 ± 0.01 c
Ledol	25.834	ND	0.06 ± 0.01	ND	ND
1,3-Dioxolane-4-methanol	3.58	ND	ND	0.13 ± 0.01	ND
widdrol	23.928	ND	ND	6.17 ± 0.02 a	5.31 ± 0.01 b
2-Propyl-1-heptanol	18.459	2.40 ± 0.02 d	15.13 ± 0.01 b	23.59 ± 0.02 a	2.81 ± 0.02 c
*(S)*-2-Amino-1-propanol	4.035	ND	ND	ND	0.18 ± 0.01
4-(Butylnitrosamino)-1-butanol	15.01	ND	ND	ND	0.69 ± 0.01
*Z,Z*-2,5-Pentadecadien-1-ol	16.08	ND	ND	ND	0.31 ± 0.02
Sclareol	23.626	ND	ND	ND	1.53 ± 0.02
1,3-Octanediol	7.195	ND	0.01 ± 0.01 bc	0.02 ± 0.01 ab	0.03 ± 0.01 a
Alcohols		43.85 ± 0.15 c	48.94 ± 0.04 b	58.36 ± 0.08 a	31.19 ± 0.05 d
Pentanal	5	0.08 ± 0.01 cd	0.21 ± 0.02 c	1.71 ± 0.01 b	2.91 ± 0.01 a
Heptanal	10.605	0.01 ± 0.00 c	0.39 ± 0.01 a	0.04 ± 0.01 b	0.01 ± 0.01 c
Succindialdehyde	12.835	0.14 ± 0.01 a	0.01 ± 0.01 b	ND	ND
Nonanal	15.769	0.27 ± 0.03 c	0.80 ± 0.02 b	0.80 ± 0.01 b	0.97 ± 0.03 a
5-Methylhexanal	10.863	ND	0.08 ± 0.02	ND	ND
Hexanal	8.643	ND	0.54 ± 0.02 a	0.35 ± 0.01 b	ND
Benzaldehyde	17.24	2.97 ± 0.04 d	4.41 ± 0.02 c	4.98 ± 0.02 b	5.62 ± 0.02 a
Aldehydes		3.47 ± 0.05 d	6.44 ± 0.03 c	7.87 ± 0.02 b	9.51 ± 0.01 a
8-Paradol	22.346	ND	2.76 ± 0.11	ND	ND
3,3-Dimethyl-4-methylamino-butan-2-one	4.53	ND	ND	0.31 ± 0.01	ND
2-Isopropyl-1-oxaspiro[4.4]nonan-4-one	14.96	ND	ND	ND	0.33 ± 0.01
3-Octanone	3.555	2.93 ± 0.06 a	0.41 ± 0.01 d	0.44 ± 0.01 c	0.64 ± 0.01 b
2-Heptanone	5.374	ND	ND	0.03 ± 0.01	0.26 ± 0.01
Ketones		2.93 ± 0.06 b	3.17 ± 0.11 a	0.78 ± 0.01 d	1.23 ± 0.00 c
Methyl cinnamate	21.856	14.29 ± 0.02 d	16.39 ± 0.02 c	18.50 ± 0.01 b	26.90 ± 0.02 a
*(E)*-9-Octadecenoic acid methyl ester	20.664	13.62 ± 0.02	ND	ND	ND
2,2,4-Trimethyl-1,3-pentanediol diisobutyrate	20.91	7.15 ± 0.05 c	11.04 ± 0.04 b	6.68 ± 0.02 d	12.38 ± 0.07 a
Phenyl cyanate	22.201	ND	5.57 ± 0.02	ND	ND
3-hydroxy-2,2,4-trimethylpentyl isobutyrate	20.822	ND	ND	4.39 ± 0.01	ND
1,2-Benzenediol,4-(1,1-dimethylethyl)-1,2-diacetate	24.094	ND	ND	0.72 ± 0.02	ND
Esters		35.06 ± 0.07 b	33.00 ± 0.04 c	30.29 ± 0.02 d	39.29 ± 0.07 a
2,4-Dimethylhexanedioic acid	27.195	0.08 ± 0.01	ND	ND	ND
12-Methylaminolauric acid	3.7	0.01 ± 0.00	ND	ND	0.02 ± 0.01
2-Octynoic acid	13.631	ND	0.02 ± 0.01 b	0.03 ± 0.01 b	0.08 ± 0.02 a
Tridecanoic acid	28.874	ND	0.08 ± 0.02 b	ND	0.29 ± 0.01 a
Dihydroxyfumaric acid	2.1	ND	ND	ND	0.12 ± 0.01
Acids		0.09 ± 0.01 b	0.10 ± 0.02 b	0.03 ± 0.01 c	0.51 ± 0.01 a
1-Methyldecylamine	3.293	0.07 ± 0.02 c	0.49 ± 0.01 a	0.11 ± 0.01 b	0.12 ± 0.01 b
1-Methyldodecylamine	3.973	0.02 ± 0.01	ND	ND	ND
2-Pentanamine	2.533	0.07 ± 0.02	ND	ND	ND
2-Aminononadecane	3.795	ND	0.45 ± 0.01 a	ND	0.02 ± 0.01 b
Pentadecane	10.77	ND	0.08 ± 0.01	ND	ND
1-Undecene	11.779	ND	0.23 ± 0.01 a	0.02 ± 0.01 b	ND
1,3,5,7-Cyclooctatetraene	12.283	ND	0.54 ± 0.01	ND	ND
5-methyl-1-Heptene	14.81	ND	ND	ND	1.45 ± 0.03
*CIS*-3-Dodecene	15.33	ND	ND	ND	0.12 ± 0.01
Alkenes		0.16 ± 0.02 c	1.79 ± 0.01 a	0.13 ± 0.01 d	1.70 ± 0.01 b
Heptylbenzene	18.147	4.83 ± 0.03 a	4.65 ± 0.05 b	0.38 ± 0.02 c	0.07 ± 0.01 d
Butylbenzene	11.131	ND	0.16 ± 0.03	ND	ND
n-Butylbenzene	13.323	ND	0.23 ± 0.03	ND	ND
1-Phenyl-decan	22.044	ND	ND	ND	3.10 ± 0.01
Aromatic hydrocarbons		4.83 ± 0.03 b	5.04 ± 0.01 a	0.38 ± 0.02 c	3.17 ± 0.02 d
2-Pentylfuran	11.629	0.23 ± 0.02 d	0.53 ± 0.01 c	0.87 ± 0.03 b	7.55 ± 0.01 a
Indole	27.35	ND	ND	0.10 ± 0.01	0.35 ± 0.01
3-Piperidinol	11.445	ND	0.01 ± 0.00	0.02 ± 0.01	ND
Heterocyclics		0.23 ± 0.02 d	0.54 ± 0.05 c	0.99 ± 0.01 b	7.90 ± 0.02 a
Phenol	22.33	0.63 ± 0.03 b	ND	ND	4.13 ± 0.03 a
2,4-Di-tert-butylphenol	25.174	8.76 ± 0.03 a	0.99 ± 0.01 d	1.18 ± 0.02 c	1.37 ± 0.03 b
Phenols		9.39 ± 0.02 a	0.99 ± 0.01 d	1.18 ± 0.02 c	5.50 ± 0.05 b

Each value is expressed as the mean ± SD (n = 3) of triplicate determinations. Significant differences between samples from different cooking times were analyzed using ANOVA. Means with different letters within a row are significantly different (*p* < 0.05) in different cooking times. RI: Retention index. ND: Not detected.

**Table 4:** ROAVs and odor descriptions of main volatile compounds in *T. matsutake* soup analyzed by HS-SPME-GC-MS.

Compounds	Threshold # (mg/kg)	ROAV				Odor Description *
		A	B	C	D	
1-Octanol	0.054	4.32	5.76	0.49	0.38	Mushroom, oil
1-Octen-3-ol	0.007	100.00	45.31	28.70	18.53	Earth, mushroom
3-Octanol	0.1	0.14	0.16	0.14	0.07	Mushroom, oil
Pentanal	0.008	0.49	1.74	12.71	14.87	Almond, bitter, oil
Heptanal	0.01	0.06	2.61	0.22	0.06	Citrus, green
Nonanal	0.015	0.87	3.59	3.17	2.65	Green, lemon
Hexanal	0.0075	ND	4.79	2.75	ND	Apple, fresh, green
Benzaldehyde	0.3	0.47	0.99	0.99	0.77	Bitter almond, burnt sugar, hyacinth-like
3-Octanone	1	0.14	0.03	0.03	0.03	Butter, herb
2-Heptanone	0.2	ND	ND	0.01	0.05	Blue cheese, fruit, green
Methyl cinnamate	0.011	62.11	100.00	100.00	100.00	Balsamic, strawberry
2-Pentanamine	170	<0.01	ND	ND	ND	-
2-Pentylfuran	0.0048	2.31	7.35	10.77	64.27	Butter, floral, fruit
Indole	0.5	ND	ND	0.01	0.03	Burnt, mothball
3-Piperidinol	10,000	ND	<0.01	<0.01	ND	-
Phenol	5.5	0.01	ND	ND	0.03	-

* Odor descriptions for volatile compounds were obtained from https://www.femaflavor.org/flavor-library (accessed on 12 February 2025). # Odor thresholds for volatile compounds were obtained from http://www.odour.org.uk (accessed on 12 February 2025) and the Compilations of Odour Threshold Values in Air, Water and Other Media. Take the average of the relative content to calculate. ND: Not detected. The use of “-” indicates that the odor description of the compounds was not found.

**Table 5:** VIP score table of different important compounds (VIP ≥ 1) based on HS-SPME-GC-MS of *T. matsutake* soup with different cooking times.

Levels	Compounds	VIP
1	1-Octen-3-ol	1.51387
2	Methyl cinnamate	1.35069
3	1-Octanol	1.23024
4	2-Pentylfuran	1.18552

## Data Availability

The original contributions presented in this study are included in the article. Further inquiries can be directed to the corresponding author.
